# Sensitivity of RT-PCR method in samples shown to be positive for Zika virus by RT-qPCR in vector competence studies

**DOI:** 10.1590/1678-4685-GMB-2016-0312

**Published:** 2017-05-22

**Authors:** Marcelo Henrique Santos Paiva, Duschinka Ribeiro Duarte Guedes, Walter Soares Leal, Constância Flávia Junqueira Ayres

**Affiliations:** 1 Centro Acadêmico do Agreste, Universidade Federal de Pernambuco, Caruaru, PE, Brazil; 2 Departamento de Entomologia, Instituto Aggeu Magalhães, Fundação Oswaldo Cruz-Pernambuco, Recife, PE, Brazil; 3 Department of Molecular and Cellular Biology, University of California-Davis, Davis, CA, USA

**Keywords:** ZIKV, RT-PCR, Aedes aegypti, vector competence

## Abstract

Tissue samples from mosquitoes artificially infected with Zika virus and shown to be positive by RT-qPCR were reexamined by RT-PCR. Using these samples we compared the two methods employed in virus RNA detection for vector competence studies. Results demonstrated that, albeit useful, RT-PCR gave false negatives with low viral loads (< 10^6^ RNA copies/ml).

Zika virus (ZIKV) is a mosquito-borne pathogen from the Flaviviridae family, which is maintained in enzootic cycles in sylvatic environments. It was first isolated in 1947 in Uganda - Africa, from a sentinel rhesus monkey (Dick *et al.*, 1952). The virus remained confined to certain areas of the African and Asian continents for the following 60 years, until in 2007 and 2013 outbreaks were reported in Yap state, Micronesia, and in French Polynesia, respectively (Lanciotti *et al.*, 2008; Cao-Lormeau *et al.*, 2014). In 2014, ZIKV was first detected in sera from eight patients from Rio Grande do Norte, a state of the northeastern region of Brazil (Zanluca *et al.*, 2015). This region quickly became the epicenter of the ZIKV epidemics observed in the country. Besides the broad panel of flavivirus-related symptoms, some Brazilian localities experienced an increase in neurological conditions, such as microcephaly, caused by ZIKV infection during pregnancy, and Guillain-Barré syndrome, associated to the human infection to this virus (WHO, 2016). Zika virus has been isolated from several mosquitoes from the genus *Aedes*, such as *Aedes aegypti*, *Ae. albopictus* and *Ae. furcifer* (Diallo *et al.*, 2014; Grard *et al.*, 2014; Brito *et al.*, 2016). Consequently, *Aedes* mosquitoes have been incriminated as the primary ZIKV vectors. However, this virus has also been isolated from *Culex quinquefasciatus, Anopheles coustani* and many other mosquito species in nature (Diallo *et al.*, 2014; Guedes *et al.*, 2016), thus the range of natural hosts and vectors could expand through the virus evolution. As the virus spread rapidly through the Americas, the World Health Organization (WHO) declared that ZIKV infection, associated with multiple neuropathologies, constituted a Public Health Emergency of International Concern (WHO, 2016). In order to properly respond to this pandemic, diagnostic tests should be broadly available and validated (Waggoner and Pinsky, 2016). Methodologies employed for ZIKV diagnosis were designed to test in human samples. An anti-Zika IgM Elisa test was established by the CDC (Centers for Disease Control and Prevention) during the Yap epidemic in 2007, however, serological data itself proved to be untrustworthy due to extensive cross-reactivity with other flaviviruses (Lanciotti *et al.*, 2008). ZIKV RNA detection by standard (RT-PCR) and quantitative RT-PCR (RT-qPCR) are currently the most reliable and rapid methods used for ZIKV diagnosis (Faye *et al.*, 2013). ZIKV RT-qPCR is faster, more sensitive and more specific assay, but the cost may be prohibitive, particularly for the analysis of large numbers of samples as in mosquito surveillance programs. In order to compare the two techniques, we performed conventional RT-PCR using samples from mosquitoes previously infected with ZIKV for which RT-qPCR data was already available (Guedes *et al.*, 2016).

A previous study using an RT-qPCR assay demonstrated that *Ae. aegypti* and *Cx. quinquefasciatus* mosquitoes are susceptible, replicate and release ZIKV through their saliva (Guedes *et al.*, 2016). Since most laboratories are equipped with standard RT-PCR platforms and ZIKV screening in wild mosquitoes requires the analysis of large samples, our study aimed at comparing the ZIKV detection limit for RT-PCR in mosquito samples previously assayed by RT-qPCR (Guedes *et al.*, 2016).

For comparison purposes, the present study grouped these samples according to cycle threshold (Ct) ranges, previously obtained by RT-qPCR (Table 1). A total of 138 samples were tested by RT-PCR using 1U High Fidelity Platinum *Taq* polymerase (Invitrogen^®^), 0.5 mM dNTPs (Invitrogen^®^), 2.5 mM of each primer (Lanciotti *et al.*, 2008), 1.5 mM of MgCl_2_ and 5 μL (~100 ng/μL) of RNA. PCR conditions were: 50 °C for 1 h, one cycle of 95 °C for 3 min, 55 °C for 15 s, 72 °C for 30 s, followed by 35 cycles of 95 °C for 15 s, 55 °C for 15 s and 72 °C for 30 s, and a final extension step at 72 °C for 10 min. PCR products were separated on 1.5% agarose gels, stained with ethidium bromide and visualized under an UV light. The strength of association between the independent variable and the dependent variable was expressed by the Odds Ratio (OR) with a 95% confidence interval. OR calculations were based on logistic regression. The statistical analysis and graph construction were done usingthe statistical language R 3.3.2 (R Core Team, 2008).

**Table 1:** Detection limits of the RT-PCR technique using mosquito positive samples artificially blood-fed with Zika virus and previously screened by RT-qPCR.

	RT-PCR detection							
Cycle threshold	Yes		No		OR	CI 95%		p-value
	N	%	N	%		Inferior	Superior	
< 32	55	88.71	4	5.26	1.00			
≥ 32	7	11.29	72	94.74	0.01	0.00	0.02	0.00

N, number; %, percentage; OR, Odds Ratio; CI, Confidence Interval.

The dramatic spread of ZIKV observed in the Americas and its implication in multiple neurological conditions have led the scientific world into an intense search for understanding different aspects of the virus, such as identification and characterization of ZIKV lineages, virus evolution, pathogenesis and molecular basis in humans, vector competence of mosquito species and identification of risk areas by vector competence studies. Thus, our study compared the sensitivity of a standard RT-PCR to a RT-qPCR assay in detecting ZIKV in mosquito samples previously infected with the virus.

The data obtained here showed that conventional RT-PCR is able to detect ZIKV load in most of the samples with Cts ranging from 17 to 31.9 (an estimated of 10^6^ to 10^10^ RNA copies/mL). However, when samples with Cts ≥ 32 were tested (less than 10^5^ RNA copies/mL), the sensitivity of the RT-PCR assay was significantly decreased (Figure 1, Table 1). Indeed, RT-PCR was employed to detect ZIKV RNA in two independent studies conducted in human samples from Brazil (Campos *et al.*, 2015; Zanluca *et al.*, 2015), despite the fact that average viremia in humans ranges from 10^3^ - 10^6^ RNA copies/mL (Lanciotti *et al.*, 2008). This same technique was also used to test the vector competence for ZIKV in *Culex* mosquitoes (Huang *et al.*, 2016).

**Figure 1:**
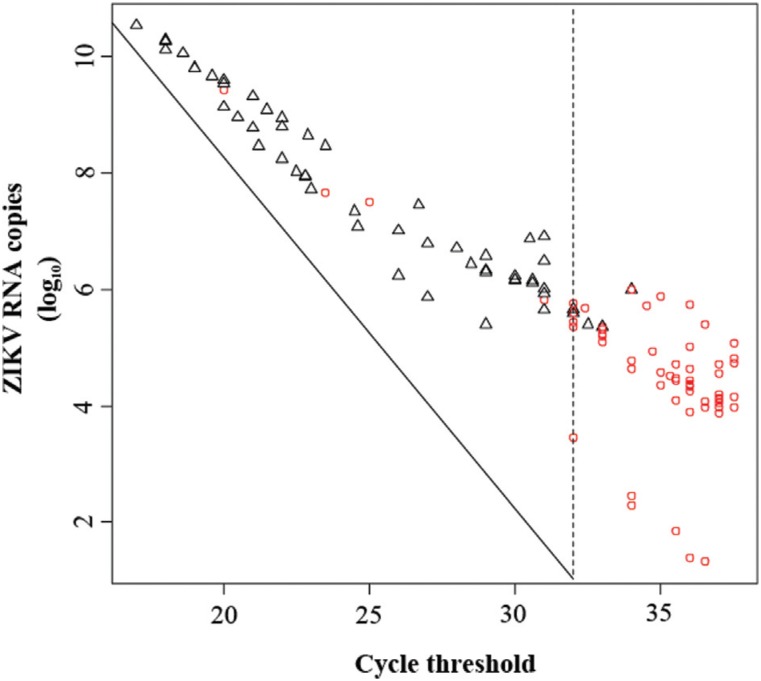
Sensitivity of standard RT-PCR for the detection of ZIKV RNA in mosquito samples previously screened by RT-qPCR.

According to our results, RT-PCR has its values. However, negative samples could represent false-negative results and consequently underreported cases of ZIKV infection. In addition, Corman *et al.* (2016) had shown that available RT-qPCR assays present limitations in clinical diagnosis. These authors concluded that, even among the RT-qPCR methods, some published assays are of limited utility within the actual ZIKV outbreak due to several factors, including low sensitivity in virus detection. With this in mind, it is more prudent to use a more sensitive technique and to assume that there is a greater chance of false-negative data in negative results obtained from ZIKV RT-PCR.

The use of Real-Time RT-PCR is currently the most adequate method for ZIKV RNA detection, both in human and in mosquito samples, even though at a higher cost (equipment and reagents), especially in large scale studies (*e.g.*, ZIKV identification in field-caught mosquitoes). Since most laboratories located in areas with active ZIKV transmission are set up with an RT-PCR platform, results presented here indicate that accessible molecular tools, such as ZIKV RT-PCR assays, are still a suitable cost effective method for a first screening of large sample sizes and are of help in the detection of of ZIKV positive samples. Consequently, this approach may provide early information on ZIKV circulation in mosquitoes prior to outbreaks of the disease. This method was recently employed by Guerbois *et al.* (2016), who screened 472 mosquito females and confirmed for the first time ZIKV transmission by *Ae. aegypti* in Mexico. However, due to its detection limits, results from ZIKV RT-PCR assays may not be able to detect ZIKV in samples with low virus titers, so negative results from RT-PCR should be re-analyzed by RT-qPCR to avoid false negatives.

In order to achieve a deeper knowledge on the ZIKV transmission cycle, the development of more affordable and accessible molecular tools is critical. This will facilitate ZIKV detection in a greater number of mosquito samples and a faster response to future outbreaks. In addition, it is important to point out that mosquitoes play a key role in the isolation of ZIKV and information on ZIKV genetic diversity. Virus isolation from human samples is extremely difficult and most isolates actually came from existing mosquito samples. Thus, earlier detection from mosquito samples would allow to isolate ZIKV strains circulating in different environments. Finally, the availability of cost effective assays, such as RT-PCR, allows to include more research groups in the fight against Zika epidemics. A more widespread use of RT-PCR may lead to the identification of other urban and wild mosquito vectors in Latin America and elucidate whether transovarian ZIKV transmission occurs in the field.
